# Realist Approach to Continuing Training of Physical Assessment Education to School Nurses (Yogo Teachers) in Japan

**DOI:** 10.7759/cureus.70026

**Published:** 2024-09-23

**Authors:** Seiji Yamashiro, Keiichiro Kita, Shinpei Fukuda, Koichiro Gibo

**Affiliations:** 1 General Medicine, Nishizaki Hospital, Itoman, JPN; 2 General Medicine, Toyama University Hospital, Toyama, JPN; 3 General Medicine, Asahi General Hospital, Asahi, JPN; 4 Internal Medicine, Nishizaki Hospital, Itoman, JPN

**Keywords:** community of practice, trust from students/parents, improved responsiveness, physical assessment, school nurses, realist approach

## Abstract

Background: As part of efforts to revitalize regional medical care, we implemented training in physical assessment for nurses and school nurses. We conducted a questionnaire survey using a realist approach to evaluate the training for school nurses that has been ongoing for 12 years.

Materials and methods: The summer training held by the Toyama Prefecture School Nurse Association includes education on severity assessment (evaluation of consciousness and vital signs), auscultation (heart, respiratory, and bowel sounds), the Heimlich maneuver, a review of Basic Life Support, and case studies. Following the training, we conducted an online questionnaire survey with participating school nurses to assess the outcome of the program.

Results: A total of 122 people responded (response rate: 35.6%) with 109 (89%) indicating that the results of the training session met or exceeded their expectations. The most common outcome of the training was improvement in their own response capabilities, followed by increased trust in students/guardians and school staff members. The training also fostered a sense of responsibility among the participants, motivating them toward further self-improvement. Furthermore, within the school, it led to collaboration with other staff members, a review of the emergency system, and improvement of equipment, which was beyond our expectations. The findings suggest that continuous repetition of basic physical assessment education will form a community of practice and bring about collaboration and revitalization within the organization as well as the acquisition of knowledge and skills.

Conclusions: Continuous training improved their ability to respond, earned the trust of students/parents and school staff, and motivated them to continue improving themselves. Furthermore, a community of practice was formed within the organization by continuing the training sessions.

## Introduction

Physical assessment education has been introduced in nursing education since the student stage; however, in school nursing education, it is still under discussion in Japan. Furthermore, there is little continuous training after graduation. The background to the introduction of physical assessment education for school nurses is as follows.

In 2004, Nanto City in Toyama Prefecture experienced a shortage of doctors and nurses following municipal mergers and the start of a new graduate clinical training system. Consequently, Nanto City asked the author to begin activities to revitalize local medical care. The doctor's group established a training program at the city hospital, the resident group engaged in health promotion activities to revitalize the community, and the city government promoted cooperation among residents, the government, and medical professionals. In 2010, a visiting nurse group launched a study group and began educating nurses about physical assessments [1].

Subsequently, the physical assessment training for nurses was continued 15 times. In the early days, training was published in newspapers, and a group of school nurses in the Toyama Prefecture expressed a desire to receive physical assessment training. The author accepted this request and initiated a physical assessment training for school nurses in 2012. In 2013, physical assessment training was introduced during a summer training session conducted by the Toyama Prefecture School Nurse Association. In addition to the summer training sessions, requests were received from schools, and physical assessment training was held 18 times over 12 years. Furthermore, since 2015, the author has been in charge of physical assessment education in the graduate course of the Department of Nursing at Toyama University and has also cooperated with specialized education. These efforts have led to the introduction of the Nurse Practitioner training course at Toyama University in 2023.

The training content for school nurses in physical assessment education includes severity assessment (state of consciousness and vital signs), auscultation (heart, respiratory, and bowel sounds), the Heimlich maneuver, the basics of Basic Life Support (BLS), and case studies. Each training session lasts two hours and 30 minutes. The lecture content on severity assessment, auscultation, the Heimlich maneuver, and the basics of BLS is the same each time, but the case studies vary each time.

This study conducted a questionnaire survey with school nurses who participated in six summer training sessions (in 2013, 2016, 2017, 2019, 2021, and 2023) organized by the Toyama Prefecture School Nurse Association to investigate the results of the training sessions. We examined the backgrounds of their participation in the sessions, which was useful for the training content and results. We also evaluated the success factors that led to these results using a realist approach.

## Materials and methods

The Toyama Prefecture School Nurse Association had 343 members. The number of participants in the six training sessions was only recorded, and no list was kept. Therefore, all members were surveyed.

The questionnaire was distributed to the mailing list of school nurses who attended the training session of the Toyama Prefecture School Nurse Association, and an online survey was conducted. The study's purpose and consent for participation were explained and confirmed within the questionnaire.

The realist approach is a methodology for qualitative research and the theory-driven evaluation advocated by Pawson and Tilley operating within the frameworks of context-mechanism-outcome. The context-mechanism-outcome configuration is presented in Table 1. This context describes in what circumstances and for whom. The mechanism is a framework for evaluating the type of intervention performed and how the outcomes were obtained [2,3].

**Table 1:** CMO configuration CMO: context-mechanism-outcome

CMO	A CMO configuration is a proposition stating what it is about a program which works for whom and in what circumstances.
Context	Realist evaluators seek to understand "for whom and in what circumstances" a program works through the study of contextual conditions.
Mechanism	Realist evaluators seek to understand "why" a program works through an understanding of the action of mechanisms.
Outcomes	Outcomes provide the key evidence for the realist evaluator in any recommendation to mount, monitor, modify, or mothball a program.

The survey questions were categorized as follows: attributes (gender, age, affiliations, years of experience as a school nurse, nursing qualifications, number of training sessions attended). Furthermore, we surveyed the reasons for attending the training session (context: background), what they learned in the training session (mechanism), and the results they achieved (outcomes) based on the realist approach. In addition, the participants were asked to provide specific examples of the results in a free-form section (see Appendices). 

As an outcome of the training, the relationship between the expectations for the training and the attributes was quantitatively evaluated.

## Results

There were 122 responses, yielding a response rate of 35.6% (122 responses/343 members). Characteristics of the respondents are presented in Table 2. All respondents were female, with 34 in their 20s (27.9%), 49 in their 30s (40.1%), 23 in their 40s (18.9%), and 16 over the age of 50 (13.1%). The affiliations were elementary school (61 respondents, 50%), junior high school (27 respondents, 22.1%), high school (24 respondents, 19.7%), special needs school (nine respondents, 7.4%), and unknown (one respondent, 0.8%). The number of years of experience as a school nurse was 1-9 years (one respondent, 0.8%), 10-19 years (21 respondents, 17.2%), 20-29 years (32 respondents, 26.2%), 30-39 years (36 respondents, 29.6%), and 40 years or more (32 respondents, 26.2%). Sixty-eight people (55.7%) had a nursing license, and 54 (44.3%) did not. The number of times participants attended the six training sessions organized by the school nurse association varied: 83 respondents (68.1%) attended once, 35 respondents (28.7%) attended twice, two respondents (1.6%) attended three times, and two respondents (1.6%) attended four times. 

**Table 2:** Characteristics of the respondents

Characteristics of the respondents (122) (response rate: 35.6%)		Number (%)
Gender	Female	122 (100)
	Male	0 (0)
Age	20s	34 (27.9)
	30s	49 (40.1)
	40s	23 (18.9)
	50 or older	16 (13.1)
Affiliations	Elementary school	61 (50)
	Junior high school	27 (22.1)
	High school	24 (19.7)
	Special needs school	9 (7.4)
	Unknown	1 (0.8)
Years of experience as a school nurse	1-9 years	1 (0.8)
	10-19 years	21 (17.2)
	20-29 years	32 (26.2)
	30-39 years	36 (29.6)
	Over 40 years	32 (26.2)
Nursing qualifications	Yes	68 (55.7)
	No	54 (44.3)
Number of training sessions attended	Once	83 (68.1)
	Twice	5 (28.7)
	Three times	2 (1.6)
	Four times	2 (1.6)

In terms of context (background), when asked about the reasons for their participation, the most common answer was acquiring physical assessment knowledge and skills to improve their judgment and response abilities. When asked who they participated for, the most common answer was for their students, followed by themselves (Table 3).

**Table 3:** CMO of participants CMO: context-mechanism-outcome

		Number of comments (%)
(1) The context of your activities		
a. In what circumstances have you participated? Why and how?	Acquire knowledge and skills to improve my judgment and ability to respond	98 (80.3)
	I want to improve my expertise as a nurse	12 (9.8)
	This is necessary	11 (9)
b. For whom?	Students	93 (76.2)
	Myself	87 (71.3)
	School	18 (14.8)
(2) Mechanisms of your activities		
a. What worked?	Heart and breath auscultation	40 (32.8)
	Severity assessment	31 (25.4)
	Heimlich maneuver	22 (18)
	Assessment of vital signs	16 (13.1)
	Emergency cases and simulations	6 (4.9)
	Management of heat illness	5 (4.1)
b. What have you studied other than the program?	Buying books and CDs	32 (26.2)
	Participation in other training courses	17 (13.9)
	Read literature and related articles	7 (5.7)
	Presentation during the training session	3 (2.5)
(3) Outcomes of activities		
a. What was the outcome?	Improved responsiveness	65 (53.2)
	Trust by students/parents and other teachers	19 (15.6)
	Review of emergency contact systems	15 (12.3)
	Self-learning	7 (5.7)
	Improved response to heat illness	4 (3.3)
b. What were the effects of your activities?	I gained confidence and politeness	46 (37.7)
	Raising staff awareness of the importance of emergency management and cooperation	17 (13.9)
	Installation of stethoscopes and pulse oximeters	13 (10.7)
(4) Rating assessment of the program	Very much below expected	2 (1.6)
	Slightly below expected	11 (9)
	Expected	71 (58.2)
	Slightly more than expected	24 (19.7)
	Very much more than expected	14 (11.5)
(5) In what areas did you make the greatest effort?	Self-learning (purchasing books and attending seminars)	38 (31.1)
	Courteous services	33 (27)
	Reflections and case conferences	11 (9)
	Cooperation among staff members	11 (9)
(6) What was your motivation for your activities?	Responsibility to protect students	58 (47.5)
	Appreciation from students/parents and staff	18 (14.8)
	Importance of emergency management and cooperation	16 (13.1)
	Horizontal connections among school nurses	3 (2.5)

When asked what they found particularly useful, they chose auscultation of the heart and respiratory sounds and assessed the severity of the illness. Outside of training, many participants engaged in self-study by purchasing books and CDs (Table 3).

**Figure 1:**
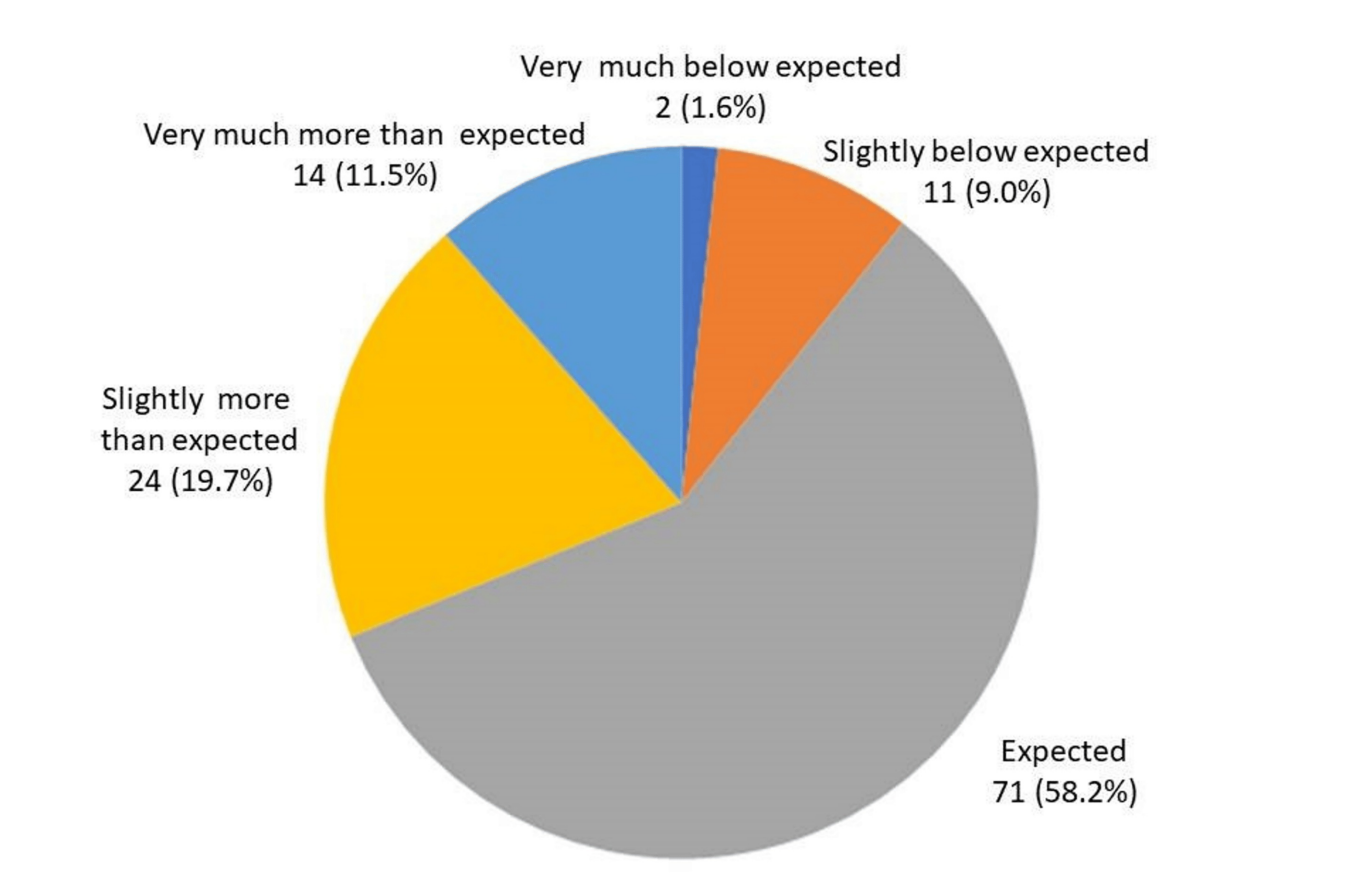
Expected of training: number (%) of total number (122)

A total of 109 (89%) of participants said that the training session met or exceeded their expectations (Table 3). Figure 1 shows all numbers and percentages regarding the rating assessment of the program. However, there were no differences in the relationship between the program's effects and the participants' attributes (Table 4).

**Table 4:** No differences between the rating assessment of the program and the participants' attributes

Participants' attributes		Expected value of training					P-value
		Very much below expected	Slightly below expected	Expected	Slightly more than expected	Much more than expected	
Number of people (%)		2 (1.6)	11 (9)	71 (58.2)	24 (19.7)	14 (11.5)	
Age (%)	20s	0 (0)	1 (9.1)	8 (11.3)	4 (16.7)	3 (21.4)	0.561
	30s	0 (0)	4 (36.4)	13 (18.3)	3 (12.5)	3 (21.4)	
	40s	1 (50)	4 (36.4)	33 (46.5)	6 (25)	5 (35.7)	
	Over 50s	1 (50)	2 (18.2)	17 (23.9)	11 (45.8)	3 (21.4)	
Affiliation (%)	Elementary school	1 (50)	5 (45.5)	37 (52.1)	9 (37.5)	9 (64.3)	0.166
	Junior high school	1 (50)	3 (27.3)	17 (23.9)	5 (20.8)	1 (7.1)	
	High school	0 (0)	2 (18.2)	11 (15.5)	9 (37.5)	2 (14.3)	
	Special needs school	0 (0)	0 (0)	6 (8.5)	1 (4.2)	2 (14.3)	
	Others	0 (0)	1 (9.1)	0 (0)	0 (0)	0 (0)	
Years of experience (%)	2-9 years	1 (50)	2 (18.2)	19 (26.8)	5 (20.8)	5 (35.7)	0.966
	10-19 years	0 (0)	5 (45.5)	22 (31)	6 (25)	3 (21.4)	
	20-29 years	0 (0)	3 (27.3)	17 (23.9)	8 (33.3)	4 (28.6)	
	30-39 years	1 (50)	1 (9.1)	12 (16.9)	5 (20.8)	2 (14.3)	
	Over 40 years	0 (0)	0 (0)	1 (1.4)	0 (0)	0 (0)	
Registered nurse (%)		0 (0)	6 (54.5)	41 (57.7)	15 (62.5)	6 (42.9)	0.402
Number of participants (%)	Once	2 (100)	9 (81.8)	44 (62)	19 (79.2)	9 (64.3)	0.392
	Twice	0 (0)	1 (9.1)	25 (35.2)	5 (20.8)	4 (28.6)	
	Three times	0 (0)	1 (9.1)	1 (1.4)	0 (0)	0 (0)	
	Four times	0 (0)	0 (0)	1 (1.4)	0 (0)	1 (7.1)	

Many improved their own response capabilities, gained the trust of students/guardians and school staff members, and reviewed the emergency contact system. Specific examples of improved response capabilities include comments on how useful it was in dealing with hyperventilation syndrome, using stethoscopes on students with asthma, installing pulse oximeters, responding to heat stroke, and deciding whether to transport a student to the hospital by ambulance (Table 3).

Changes in themselves and those around them included more polite responses, increased confidence, improved awareness of the importance of crisis management and cooperation among staff members, and installation of items such as stethoscopes and pulse oximeters. To achieve these outcomes, participants have initiated improvements in their practice. Motivations driving these changes included a sense of responsibility for protecting students, trust and gratitude among students/guardians and staff members, and improved staff awareness of the importance of crisis management and cooperation (Table 3).

## Discussion

A total of 109 (89%) school nurses rated the physical assessment training as meeting or exceeding expectations. We expected the evaluation of the training to differ depending on the attributes of the school nurse (years of experience, nursing qualifications, etc.); however, there was no difference. This may be due to the lack of educational opportunities in the field and the increasing role of school nurses in the field, regardless of the years of experience.

It has been reported that education in physical assessment and urgency and severity assessment is important for school nurse training institutions [4,5]. However, there are some differences between nursing schools and other training institutions, with symptom- and body part-specific assessments carried out more often by nursing schools [6]. Practical education and training in symptom- and body part-specific assessments are important to reduce the sense of difficulty during the emergency treatment process at the scene [7]. School nurses use four elements in their health consultation activities (physical, psychological, social, and lifestyle assessments); physical assessments utilize psychological, social, and lifestyle assessments to understand background factors [8].

The most common outcome of the training was an improvement in their own response capabilities, followed by increased trust from students/guardians and staff members. This also motivated them (a sense of responsibility to protect students), which prompted them to further improve themselves. Furthermore, within the school, it led to greater collaboration with other staff members, a review of the emergency system, and improvement of equipment, which was beyond our expectations.

Repeated practice is necessary to improve response capabilities in the field, and it is necessary to create and practice various simulation cases [9]. It is also necessary to acquire the basis for judgment when deciding on a response [10,11]. Furthermore, clinical reasoning education is beginning to be taught in this field [12].

Nursing training institutions are making progress in providing physical assessment education. However, auscultation and percussion are rarely used in practice, and it has been reported that the involvement of educators is necessary [13]. Furthermore, some institutions are using stimulated recall interviews/reflections as a teaching method for clinical reasoning [14,15].

**Figure 2:**
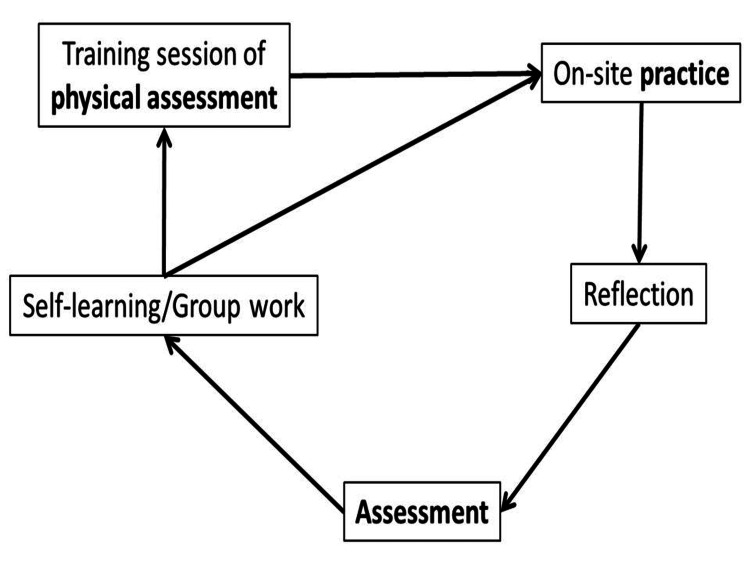
Community of practice for school nurse

This continuous training not only improved physical assessment skills but also fostered self-improvement and positively impacted other school staff members. The meaning of "continuous" does refer not only to the time spent at the training session but also to the ongoing relationship between the organizers and the author, who also supported the creation of response manuals and case studies for use in schools in addition to the training session. After the training session, the participants applied their learning into practice, engaged in reflections, and evaluated reflections, forming a community of practice through self-improvement or participation in training sessions (Figure 2). Lave and Wenger stated that a community of practice is "a group of people who share interests, problems, and enthusiasm about a certain topic and deepen their knowledge and skills in that field through sustained mutual interaction" [16,17]. Itoh et al. reported that a community of practice was formed on a business unit basis within a nursing station during a continuous training session for visiting nurses [18]. In addition, a study of communities of practice from a management perspective has been reported [19]. Thus, the formation of a community of practice promoted cooperation and revitalization within the organization, along with the acquisition of knowledge and skills, and the trust of students/guardians and other staff members led to motivation for self-improvement.

Limitations

This study involved a questionnaire survey on physical assessment training in a specific area, and no comparison was made with other similar training programs. Furthermore, the sample size was small, and no statistically significant differences were found. In addition, as this was an evaluation based on a relatively new realist approach, further research is required.

## Conclusions

We conducted a questionnaire survey on the results of a physical assessment training program for school nurses. Continuous training improved their response capabilities, earned the trust of students/parents and staff members, and motivated them to further improve themselves. Furthermore, it was possible to form a community of practices within the organization that continued to hold training sessions.

## Appendices

Questionnaire

(1) Characteristics

Gender:

Age:

Affiliations:

Years of experience:

Nursing qualifications:

Number of training sessions attended:

(2) Context of your activities

a. In what circumstances did you participate? Why and how?

b. For whom?

(3) Mechanism of your activities

a. What worked?

b. What have you studied other than the program?

(4) Outcome of your activities

a. What was the outcome?

b. What were the effects of your activities?

(5) Rating assessment of the program

1. Very much below expected

2. Slightly below expected

3. Expected

4. Slightly more than expected

5. Very much more than expected

(6) In what area did you make the most effort?

(7) What was your motivation for your activities?

(8) Free comments
